# The Role of Arginine-Phenylalanine-Amide-Related Peptides in Mammalian Reproduction

**DOI:** 10.22074/ijfs.2015.4540

**Published:** 2015-10-31

**Authors:** Mohammad Saied Salehi, Amin Tamadon, Mohammad Reza Jafarzadeh Shirazi, Mohammad Reza Namavar, Mohammad Javad Zamiri

**Affiliations:** 1 Department of Animal Science, College of Agriculture, Shiraz University, Shiraz, Iran; 2 Transgenic Technology Research Center, Shiraz University of Medical Sciences, Shiraz, Iran; 3 Histomorphometry and Stereology Research Center, Clinical Neurology Research Center, Shiraz University of Medical Sciences, Shiraz, Iran

**Keywords:** RFamide-Related Peptide, Gonadotropin Inhibitory Hormone, Reproduction, Mammals

## Abstract

Until 2000 it was believed that gonadotropin-releasing hormone (GnRH) was the sole regulator of hypophyseal gonadotropes. In 2000, the discovery of a gonadotropin inhibitory hormone (GnIH) initiated a revolution in the field of reproductive physiology. Identification of GnIH homologues in mammals, the arginine-phenylalanine-amide (RFamide)-related peptides (RFRPs), indicated a similar function. Subsequently, further works conducted in various laboratories worldwide have shown that these neuropeptides inhibit the hypothalamic-hypophyseal axis. This review discusses the role of RFRPs in mammalian reproductive processes.

## Introduction

Gonadotropin-releasing hormone (GnRH), the main stimulator of gonadotropes and secretion of gonadotropins, was first purified from the pig and sheep hypothalami in the 1970s (1, 2). For years GnRH has been considered the only regulator of the hypothalamic-hypophyseal-gonadal axis. Gonadal steroids and inhibin regulate gonadotropin secretion via negative/positive feedback mechanisms. Although existence of a hypothalamic inhibitor of gonadotropin secretion was suspected earlier (3), in 2000 researchers discovered a 12 amino acid peptide (SIKPSAYLPLRFamide) in the quail brain which could directly inhibit GnRH release. It was subsequently named the gonadotropin inhibitory hormone (GnIH) (4). During the last 13 years, avian homologues of GnIH have been identified in several mammalian species and named argininephenylalanine- amide (RFamide)-related peptides (RFRP). In this review we describe the chemical structure, biosynthesis and functions of RFRPs related to mammalian reproduction and their possible roles in other physiologic events.

### History, biosynthesis and chemical structure of RFamide-related peptides

The RFRPs are a family of peptides with an arginine- phenylalanine (RF-NH_2_) sequence at their carboxyl terminals. Researchers have discovered the first peptide of this family in shell ganglions (FMRFamide) (5). The first RFRP in vertebrates was discovered in the avian brain (LPLRFamide) (6). In 2000 researchers reported that one of the RFRPs inhibited the secretion of gonadotropins. Since then, GnIH homologues have been identified (Table 1) in several species of mammals, including humans (7), monkeys (8), cattle (9), sheep (10, 11), rats, mice (12) and hamsters (13).

Following transcription and translation of the *RFRP* gene, a prepeptide is synthesized which routinely separates into two mature peptides, RFRP- 1 and RFRP-3 (Table 1). The carboxy terminals of RFRPs contain a sequence of leucine-proline-XXX-arginine-phenylalanine (LPXRF, X=leucine or glutamine) followed by glycine (G) as an amidation signal, and arginine (R) or lysine (K) that act as endoproteolytic basic amino acids (14). However, in humans, monkeys, and cattle, RFRP-2 is also built from a prepeptide which differs from LPXRF. This prepeptide contains RS-amide sequences or an RSamide in the carboxyl terminal. RFRP-1 and RFRP-3 bind the same receptor, named GPR147 (also known as OT7T022 and NPFF-1) with similar affinity (15).

**Table 1:** Amino acid sequence of the RFamide-related peptide (RFRP) prepeptide in different mammalian species


Species	Aminoacidsequence	No.ofaminoacids

Human	MEIISSKLFILLTLATSSLLTSNIFCADELVMSNLHSKENYD-KYSEPRG	49
Monkey	MEIISSKLFILLTLATSSLLTSNISCADELMMSSLHNKENYD-KYSEPRG	49
Cow	MEIISLKRFILLMLATSSLLTSNIFCTDESRMPNLYSKKNYD-KYSEPRG	49
Sheep	MEIISLKRFILLMLATSSLLTSNIFCTDESRIPSLYSKKNYD-KYSEPRG	49
Rat	MEIISSKRFILLTLATSSFLTSNTLCSDELMMPHFHSKEGYG-KYYQLRG	49
Mouse	MEIISLKRFILLTVATSSFLTSNTFCTDEFMMPHFHSKEGDG-KYSQLRG	49
Hamster	MEIISSKRFILLTLATSSLLTSN IFCTEELMMPHFHS KE KED-KYSQPTG	49

Human	YP--KGERSLNFEELKDWGPKNVIKMSTPAVNKMPHSFANLPLRFGRNVQ	97
Monkey	YP--KRERSLNFEELKDWGPKNVIKMSTPAVNKMPHSVTNLPLRFGRTTE	97
Cow	DLGWEKERSLTFEEVKDWAPK--IKMNKPVVNKMPPSAANLPLRFGRNME	97
Sheep	DLGWEKERSLTFEEVKDWGPK--IKMNTPAVNKMPPSAANLPLRFGRNME	97
Rat	IPKGVKERSVTFQELKDWGAKKDIKMSPAPANKVPHSAANLPLRFGRNIE	99
Mouse	IPKGEKERSVTFQELKDWGAKNVIKMSPAPANKVPHSAANLPLRFGRTID	99
Hamster	ISKGEKERSVSFQEVKDWGAKNVIKMSPAPANKVPHSAANLPLRFGRTLE	99
RFRP-1		
Human	EERSAGATANLPLRSGRNMEVSLVRRVPNLPQRFGRTTTAKSVCRMLSDL	147
Monkey	EERSTGAIANLPLRSGRNMEVSLVRQVLNLPQRFGRTTTAKSVCRTLSDL	147
Cow	EERSTRAMAHLPLRLGKNREDSLSRWVPNLPQRFGRTTTAKSITKTLSNL	147
Sheep	EERSTRVMAHLPLRLGKNREDSLSRRVPNLPQRFGRTIAAKSITKTLSNL	147
Rat	DRRSPRARA-----------------NMEAGTMSHFPSLPQRFGRTT-ARRITKTLAGL	140
Mouse	EKRSPAARV------------------NMEAGTRSHFPSLPQRFGRTT-ARS-PKTPADL	139
Hamster	EDRSTRART-------------------NMEARTLSRVPSLPQRFGRTT-ARSIPKTLSHL	140
RFRP-3		
Human	CQGSMHSPCANDLFYSMTCQH-QEIQNPDQKQSRRLLFKKIDDAELKQEK	196
Monkey	CQGSLHSPCANDLFYSMTCQH-QEIQNPDQKRSRRLVFQKMDDAELKQEK	196
Cow	LQQSMHSPSTNGLLYSMACQP-QEIQNPGQKNLRRRGFQKIDDAELKQEK	196
Sheep	LQQSMHSPSTNGLLYSMTCRP-QEIQNPGQKNLRRLGFQKIDDAELKQEK	196
Rat	PQKSLHSLASSELLYAMTRQH-QEIQSPGQEQPRKRVFTETDDAERKQEK	189
Mouse	PQKPLHSLGSSELLYVMICQH-QEIQSPGGKRTRRGAFVETDDAERKPEK	188
Hamster	LQRFLHSMATSEVLNAMTCQH-GEIQSPGGKQPRRQAFMETDDEEGKHEK	189
Rat	IGNLQPVLQGAMKL	203


### Extension of RFamide-related peptide neuronal bodies and fibers in the mammalian brain

RFRP neuronal bodies have been detected in the rat dorsomedial hypothalamus and proven by a number of research studies using different antibodies. These antibodies included an antibody produced against the sparrow GnIH produced in rabbits (16) and an antibody produced against the sequence 119-132 of prepeptide RFRP (17). We also reported similar findings in rats (18) by using an antibody against the quail GnIH produced in rabbits (supplied kindly by Professor K. Tsutusi). The same neuronal extensions were also found in the brains of hamsters (13, 16) and mice (16). In sheep, neurons that expressed RFRP were identified in the dorsomedial hypothalamic area (DMH), paraventricular nucleus (PVN), the area between these nuclei (11) and the preoptic area (POA) (19). We showed that agouti-related peptide (AgRP) and RFRP coexpressed in 19 to 32% of the arcuate (Arc) neurons during various phases of the estrous cycle in the ewe (20). In addition, we observed similar neuronal extensions in the brains of native Fars goats (21). Positive cells were found in the monkey periventricular nuclei (8) and human DMH (7).

In rodents, fibers and terminals of RFRP neurons were observed in the middle areas of the brain, limbic areas (POA, septal and amygdala), rostral hypothalamus and Arc (13, 16). In the monkey brain, RFRP fibers were observed in most parts of the brain, including the hemispheres or telencephalon, septal nuclei and accumbens, hypothalamus and particularly POA, Pe, PVN and ARC, habenular nuclei, thalamus, upper calculi of the midbrain, Raphe nuclei and the pons (8).

### The inhibitory role of RFamide-related peptides

In all vertebrate species studied from fish (22) to humans, the RFRP/GnIH peptides decreased the secretion of gonadotropins, particularly luteinizing hormone (LH), via actions on GnRH neurons and/ or gonadotropes. This showed the possibility of a protective role in various species (16, 23, 24). Recently published reports indicated that these peptides in certain situations did not affect LH secretion or even have a stimulatory effect, which in the following they also be explained.

### The inhibitory role of RFamide-related peptides

In all vertebrate species studied from fish (22) to humans, the RFRP/GnIH peptides decreased the secretion of gonadotropins, particularly luteinizing hormone (LH), via actions on GnRH neurons and/ or gonadotropes. This showed the possibility of a protective role in various species (16,23,24). Recently published reports indicated that these peptides in certain situations did not affect LH secretion or even have a stimulatory effect, which in the following they also be explained. 

### The effect of RFamide-related peptides on the gonadotropin-releasing hormone neuronal system

Any direct effect of RFRP on GnRH neurons necessitates a direct connection between RFRP neuronal terminals and GnRH neurons. In the POA of male rats, research has shown that RFRP fibers formed a close association with approximately 75% of GnRH neuronal bodies (24). Similar finding was reported in female hamsters (more than 40%), mice and rats (16). In another study, there was communication of RFRP fibers with GnRH neurons observed in the anterior hypothalamic area, MBH (approximately 30%) and POA of sheep (25). In sheep, co-expression of RFRP and GnRH during proestrus and estrus (follicular phase) and the luteal phase has been reported. During the luteal phase of sheep, more POA neurons expressed RFRP compared to the follicular stage, while there were no differences in the number of GnRH neurons in the hypothalamus, which indicated a direct effect of RFRP neurons in POA on GnRH neurons and an indirect effect on LH secretion (23). 

In the POA of monkeys, 67.9% of GnRH neurons established connections with RFRP fibers (8), with similar connections observed in the human brain (7). More than 80% of GnRH neurons of POA in the Siberian hamster expressed GPR147 receptor (13). In adult male and female diestrus mice, there was a close relation between RFRP-3 neuron terminals with 25% of the body of GnRH neurons in the medial septum and 27% in the rostral part of the POA, and 33% of GnRH neurons which expressed *GPR147* mRNA (26). Addition of RFRP into GnRH neurons *in vitro* decreased the firing rate of 41% of the neurons. However, electrophysiologic evaluations in that study showed that RFRP treatment had a stimulatory effect on 12% of the neurons and no effect on 47% of neurons (27). In the same study, RFRP treatment caused hyperpolarization of more than 50% of GnRH neurons (28). 

Intraventricular administration of RFRP rapidly decreased plasma LH concentration in male rats (24), ovariectomized hamsters (16) and Siberian hamsters maintained on a long-day photoperiod; however, injection of RFRP in hamsters on a shortday photoperiod stimulated LH release 30 minutes after the injection (13). In another research, intraventricular injection of RFRP-3 stimulated expression of c-Fos in GnRH neurons and increased both LH and testosterone secretion (29). In contrast, intraventricular injection of RFRP in ovariectomized rats had no effect on the mean plasma LH concentration or frequency of LH pulses (30). Intraventricular injection of RFRP in ovariectomized rats following induction of the GnRH/LH surge by estradiol (E_2_) and progesterone decreased the activity of GnRH neurons (evaluated based on *c-Fos* gene expression) by 50 to 60% (31). However, in that study, central injection of RFRP in ovariectomized rats treated with E_2_ implant had no effect on LH pulse and amplitude or mean concentration of LH. Recently, it was observed that intraventricular RFRP-3 injection in ovariectomized ewes had no effect on plasma LH concentration (32). Intraventricular administration of RF9, a potent and specific antagonist of the RFRPs receptor (33), resulted in a rapid, dose-dependent increase in gonadotropin secretion in male and female rats (34). Collectively, these finding suggested that RFRP could change GnRH secretion via a direct action on the GnRH neuronal system [for more information see the review by Anderson (35)].

### Effect of RFamide-related peptides on hypophysis

In order to generate a physiologic effect on gonadotropin secretion, the hypothalamic RFRP neuronal terminals must either form a close association with GnRH neurons in the median eminence (ME) and/or RFRP receptors must be located on gonadotropes. The RFRP neuronal terminals are found in the external layer of the ME in hamsters (13,16,36), sheep (11), monkeys (8) and humans (7). GPR147 expression is reported in the hypophysis of hamsters (36), rats (37,38) and humans (7). The presence of RFRP in the hypothalamic-hypophyseal portal vein of sheep has been reported by Smith et al. (39). In rats, while some researchers did not observe RFRP fibers in ME (17,24), others reported the presence of RFRP fibers in male (40) and female (18) Sprague-Dawley rats.

Fluorogold is a retrograde tracer that does not cross the blood-brain barrier but can be absorbed from portal arterioles of hypophysis by neurons terminals in the external area of ME. Intraperitoneal injection of this tracer has been used to detect central hypophysiotropic cells. The results indicated that more than 90% of GnRH neurons and only 3 out of 234 RFRP neurons in the POA of rats stained with Fluorogold (17). 

Intravenous injection of RFRP decreased LH secretion in several mammals; however, the mode of action might differ in various species. Intravenous injection of RFRP in ovariectomized ewes decreased the amplitude of LH pulses; but had no effect on pulse frequency (11). Intravenous injection of RFRP in castrated bulls decreased the frequency of LH pulses, however a single injection had no effect (41). Intravenous injection of RFRP in ovariectomized rats (30) and ovariectomized hamsters decreased mean concentrations of LH (16). 

The addition of RFRP to cultures of hypophyseal cells of rats (30), cows (41) and sheep decreased GnRH-induced LH secretion. Interestingly, the addition of GnRH to hypophyseal cells increased expression of *LHβ* mRNA in rams (4 times) and ewes (2.5 times), but RFRP inhibited LHβ subunit expression (42). On the other hand, it was also reported that treatment with RFRP (31) or RF9 (an RFRP receptor antagonist) (34) had no effect on GnRH-induced LH secretion in a hypophyseal cell culture in rats. 

### Effect of RFamide-related peptides on gonads

In addition to expression in the brain, expression of RFRPs and their receptors in mammalian gonads have been reported. In male hamsters, cells that expressed RFRP were observed in the seminiferous tubules. Its receptor, GPR147, was observed in spermatocytes and spermatids (43). In the monkey, RFRP and its receptor were expressed in Leydig cells, spermatogonia and spermatocytes (44). RFRP was also found in the granulosa and luteal cells of mice ovaries (45). 

RFRP peptides in granulosa cells of preovulatoy follicles and corpus luteum along with GPR147 receptors in granulosa cells, theca cells and the corpus luteum have been observed in women. RFRP-3 could inhibit the effect of gonadotropins on progesterone production and expression of StAR protein (46). Thus, it was postulated that RFRP might have autocrine/paracrine roles in gametogenesis and steroidogenesis (44). 

### Effect of sex steroids on the RFamide-related peptide system

A low concentration of E_2_ secreted during the majority of the ovarian cycle in most mammals exerts a negative feedback effect on GnRH neurons by keeping GnRH/LH secretion at a basal level. During the preovulatory period, high levels of estrogen secreted from mature follicles results in a GnRH/LH surge via a positive feedback effect. The GnRH neurons do not express alpha E_2_ receptors (ER_α_) which are essential for the positive and negative feedback effects of E_2_ (47). Therefore, it seems that other steroid sensitive neurons are intermediaries of the estrogen effect on regulation of GnRH (and LH) secretion.

Approximately 40% of RFRP neurons in the brain of female hamsters (16) and 18% in ovariectomized mice expressed ER_α_ (48). Therefore, RFRP neurons might intermediate the E_2_ feedback effect. We studied the expression of *RFRP* mRNA and peptides during the estrous cycle of rats. Expression of *RFRP* mRNA in proestrus was less than in diestrus and the numbers of neurons that expressed the RFRP peptides during proestrus and early estrus was less than during estrus and diestrus. Increased secretion of E_2_ in the evening of proestrus from dominant follicles in addition to the positive feedback effect on GnRH/LH surge might facilitate GnRH/LH secretion by exerting an inhibitory effect on RFRP expression in DMH (18). In another study we evaluated the numbers of neurons that expressed RFRP in DMH/PVN during the follicular and luteal phases of goats. The numbers of positive cells in the follicular phase (periovulatory period) was less than in the luteal phase (21). Consistent with this finding, the numbers of RFRP neurons decreased during the preovulatory period in hamsters (36). It was also reported that the number of POA neurons that expressed RFRP was greater during the luteal phase compared with the follicular phase in sheep (19).

E_2_ implants (100 μg/ml) for 4 days in ovariectomized mice decreased the number of RFRP cells and the expression of *RFRP* mRNA per cell (48) as determined by in situ hybridization. Possibly, only high or long term levels of E_2_ could decrease the expression of RFRP in rodents because only once subcutaneous injection of E_2_ in ovariectomized hamsters sufficiently increased the activity of RFRP neurons (evaluated by c-Fos expression) at 3 and 6 hours after injection (16). In contrast to these findings, during the breeding and non-breeding seasons for sheep, there was no difference in the numbers of neurons that expressed *RFRP* mRNA and *RFRP* mRNA levels per cell between ovariectomized ewes and ovariectomized ewes that received E_2_ implants (25).

RFRP neurons in the brain of male hamsters expressed an androgen receptor (16), however castration of male hamsters or treatment with testosterone implants for 4 weeks had no significant effect on the number of cells that expressed RFRP (49). Therefore, more studies should be conducted to clarify the mechanism of sex steroid action on RFRP neurons.

### The effect of a photoperiod on the RFamiderelated peptide system

Reproductive activity in several mammalian species shows salient seasonal alterations due to basal alterations in secretion of reproductive hormones. In compliance with the action of RFRP mammalian reproduction, it is logical that RFRP expression in seasonal breeders will be harmonized with changes in the photoperiod. Contrary to the expectation in Syrian and/or Siberian hamsters (long-day breeders), there were fewer neurons that expressed RFRP mRNA and RFRP peptide during the short-term photoperiods (8 hours light) compared with the long-term (16 hours light) photoperiods (13,49,50). These findings were not related to the specific time of day since gene expression was the same during 24 hours (49). 

Aggregation of RFRP fibers in POA and rostral hypothalamus (aggregation area of GnRH neurons) and the percent of GnRH neurons that established connections with RFRP fibers were less during the shortterm compared to the long-term photoperiod (13,50). On the other hand, pinealectomy prevented a decrease in RFRP expression during short days (13,49). A 60day melatonin injection administered to hamsters maintained under long-term photoperiods remarkably decreased *RFRP* mRNA expression and produced the same response as in the short-term photoperiod (49). Administration of melatonin for 13 weeks to pinealectomized hamsters kept under a short-term photoperiod decreased *RFRP* gene expression (13). Therefore, melatonin appeared to decrease the activity of RFRP neurons during short-term photoperiods. 

Coordination of these findings with the inhibitory role of RFRP was difficult because when the lowest level of expression was seen, the reproductive system was inactive. In Siberian hamsters, the relative expression of *RFRP* mRNA during average days (13.5 hours light) was more than 40 times the long days (16 hours light) (51). Therefore, it was possible that a considerable increase in RFRP expression during the early period of reproductive system regression (average days) would be necessary to inhibit the reproductive axis. However, this level of expression in hamsters whose reproductive axis did not completely regress was not necessary. Because intraventricular injections of RFRP in hamsters maintained under short-term photoperiod conditions had a stimulatory effect (13) it was possible that the decrease in RFRP expression during short-term photoperiods was important for inhibition of reproduction. 

Unlike hamsters, sheep and goats are shortday breeders. The number of RFRP that expressed neurons during the non-breeding season in sheep (long-term photoperiod) was approximately 40% more than during the breeding season, but there was no difference in the number of *RFRP* mRNA per cell (25). However, in another study, *RFRP* mRNA expression was highest during the long days (10). Communication of RFRP fibers with GnRH neurons in POA and rostral hypothalamus was highest during the non-breeding season (25). In addition to the seasonal change of RFRP expression in DMH/PVN nuclei, *RFRP* mRNA expression in epithelial or ependymal cells around the ventricle was seen only in long days (10). 

Recently we evaluated RFRP expression in DMH/PVN nuclei in goats during the breeding season (follicular and luteal phases) and anestrus. In both nuclei, the number of cells that expressed RFRP was higher in during anestrus compared to the follicular phase. However, there was no difference between the anestrus and luteal phases. We also determined the number of positive neurons in the rostral, middle and caudal parts of the DMH/PVN. In the rostral areas, more RFRP neurons were observed during anestrus than during the follicular phase; however, there was no effect of the reproductive stage recorded in middle and caudal parts of these nuclei (21). These results in sheep and goats were in accordance with the inhibitory role of RFRP on the reproductive axis. 

### The probable action of RFamide-related peptides on other physiologic events

The diffuse distribution of RFRP neuronal processes in the brain is suggestive of additional roles for this neuropeptide in physiology. The RFRP neuronal terminals in sheep brain are extended to neurons of orexin, melanin, proopiomelanocortin and neuropeptide Y; therefore, RFRP neurons may have a role in the regulation of appetite and energy balance, and possibly function as a link between nutrition and reproduction (52). Long term malnutrition (2 weeks) has been shown to increase *RFRP-3* mRNA expression in DMH of the hypothalamus in ovariectomized female rats (53). 

Furthermore, in the monkey brain, RFRP fibers had a close relation with neurons of dopamine, beta-endorphin and GnRH-II. Since dopamine neurons express GPR147, it was suggested that RFRP might stimulate prolactin secretion by inhibition of dopamine neurons (8). Consistent with this idea, we showed that the numbers neurons that expressed RFRP in suckling rats (in which plasma prolactin is at its highest level) was higher than in non-suckling rats (54). Increased *RFRP-3* mRNA expressions in DMH of the hypothalamus while increasing milk production in rats might be the inhibitory factor for GnRH secretion (55). 

Based on the findings that prolactin (56,57) and oxytocin (58) secretion increased during the refractory period after ejaculation in men, we proposed a hypothesis that increased RFRP expression after ejaculation might be the cause of the post-ejaculation refractory period in men (59). Intracerebroventricular injection of RFRP in rats also increased the activity of oxytocin neurons in the hypothalamus and oxytocin concentrations in plasma. It was shown that the supraoptic and PVN nuclei of the hypothalamus expressed *GPR147* mRNA (60). Therefore, RFRP peptides might also participate in the regulation of oxytocin secretion. 

Coexpression of RFRP and AgRP in the Arc neurons of the ewe has been reported which indicated a probable role of these two peptides in control of the ewe reproductive cycle. This study also showed that ovarian steroids affected expression of these peptides in the Arc of the hypothalamus and might be a link between energy homeostasis and reproduction (23). 

### RFamide-related peptides and treatment of reproductive disorders

As mentioned before, RFRP peptides have an opposite effect against GnRH in numerous situations and inhibit secretion of gonadotropins. However in some cases they may have an effect on LH release and a stimulatory effect (please see the previous sections). GnRH analogs (agonists and antagonists) have been applied in the treatment of a wide spectrum of reproductive disorders, including precocious puberty, endometriosis, uterine fibroids, prostatic hyperplasia, prostatic and breast cancers. By 2000, more than 2 billion dollars in sales of these compounds was recorded (61). Therefore, considering the potential effect of RFRP in inhibition of gonadotropins, the use of these peptides in the future for the treatment of reproductive disorders would be expected (62). 

The inhibitory effect of stress on reproductive performance has been demonstrated. Stress leads to activation of the hypothalamus-pituitary-adrenal axis which inhibits GnRH secretion. It seems that the effects of stress on the hypothalamuspituitary-gonad axis is mediated by adrenal steroid hormones (glucocorticoids). Since neurons of GnRH do not express glucocorticoid receptors, it is possible that these steroid hormones affect neurons upstream of GnRH neurons and change the release of GnRH. Reports have shown that RFRP neurons mediate the effects of stress on reduction of GnRH/LH secretion and stop of the reproductive axis (38,63). Therefore, it is possible that using RFRP antagonists or antibodies against RFRP safeguard reproductive performance in stressful situations. Also, as noted above, increase in RFRP expression may be involved in the post-ejaculatory refractory period (59) Hence disabling the RFRP system may shorten this period. 

## Conclusion

Based on the findings in mammals, RFRPs are homologues of GnIH in birds and can inhibit LH secretion and the reproductive axis; however, their mode of action is not yet clearly established. For example it is not known whether they inhibit the GnRH system and/or have a direct effect on hypophyseal gonadotropes in preventing gonadotropin secretion. Amongst studied species, the most contradictory data have been reported in the rat. Based on extensive connection between the RFRP neurons and other neurons, more studies will be required to identify the exact role of these peptides in reproduction and other physiologic functions. 
